# Supplementary material for: Somatosensory alpha oscillations gate perceptual learning efficiency
Source: Nat Commun. 2019 Jan 16;10:263. doi: 10.1038/s41467-018-08012-0 (PMC6335466; doi:10.1038/s41467-018-08012-0)

Supplementary Information

Somatosensory alpha oscillations gate perceptual learning efficiency

Brickwedde et al. 2019

Supplementary Tables

Supplementary Table 1 | Post hoc tests – two-way mixed *ANOVA* 2 x 3 (time x condition) with raw alpha power comparing baseline day 1 with the last block of NF training;

Alpha power	Group		*Mdiff*(*SEM*)	*t*(*df*)	*p*	95% Confidence interval	
						Upper	Lower
Baseline day 1	Alpha Up	Alpha Down	.28(1.39)	.20(30)	.844	-2.567	3.120
		NF-PR	-.42(1.18)	-.35(31)	.725	-2.827	1.990
	Alpha Down	NF-PR	-.70(1.27)	-.55(29)	.587	-3.234	1.894
Last NF training day 2	Alpha Up	Alpha Down	3.26(1.63)	2.00(30)	.055	1.633	6.597
		NF-PR	.99(1.37)	.72(31)	.476	-1.808	3.788
	Alpha Down	NF-PR	-2.27(1.55)	-1.45(29)	.153	-5.443	.898

Alpha power		Group	*Mdiff*(*SEM*)	*t*(*df*)	*p*	95% Confidence interval	
						Upper	Lower
Baseline day 1	Last NF training day2	Alpha Up	-2.55(.65)	-3.94(16)	.001*	-3.930	-1.180
		Alpha Down	.43(.68)	,63(14)	.539	-1.035	1.898
		NF-PR	-1.15(.58)	-1.98(15)	.066	-2.381	.088

*Note. *The level of significance (p < .05) was obtained after Bonferroni adjustment (.05/9 =.006); Data are presented as mean ± SEM.*

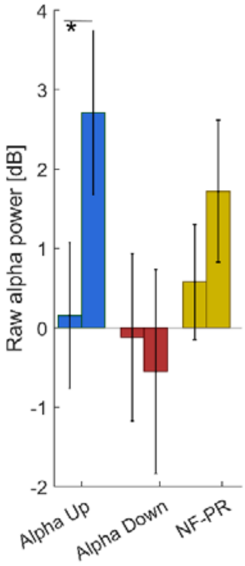

Supplementary Table 2 | Post hoc tests – Two-way mixed *ANOVA* 3 x 2 (time x condition) with alpha power during NF training on day 2, normalized on baseline day 1 in both neurofeedback groups;

Group		Alpha power day 2	*Mdiff*(*SEM*)	*t*(*df*)	*p*	95% Confidence interval	
						Upper	Lower
Alpha Up	Alpha Down	NF block 1	1.54(.63)	2.46(30)	.020	.260	2.821
		NF block 2	2.86(.72)	3.95(30)	.000**	1.378	4.331
		NF block 3	3.00(.81)	3.71(30)	.001**	1.347	4.654

Group	Alpha power day 2		*Mdiff*(*SEM*)	*t*(*df*)	*p*	95% Confidence interval	
						Upper	Lower
Alpha Up	NF block 1	NF block 2	-1.14(.33)	-3.48(16)	.003*	-1.838	-.445
		NF block 3	-1.72(.44)	-3.92(16)	.001*	-2.652	-.791
	NF block 2	NF block 3	-.58(.36)	-1.61(16)	.127	-1.344	.184
Alpha Down	NF block 1	NF block 2	.17(.28)	.61(14)	.550	-.432	.777
		NF block 3	-.26(.23)	-1.12(14)	.282	-.763	.240
	NF block 2	NF block 3	-.43(.22)	-1.95(14)	.072	-.912	.044

*Note. *The level of significance (*p < .05; ** p < .01) was obtained after Bonferroni adjustment (.05/9 =.006; .01/9 = .001); Data are presented as mean ± SEM.*

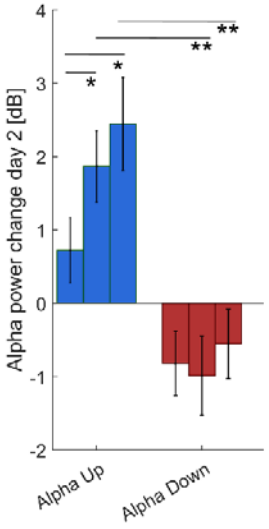

Supplementary Table 3 | Post hoc tests – Two-way mixed *ANOVA* 2 x 2 (time x condition) with alpha power during NF training on day 1, normalized on baseline day 1 in both neurofeedback groups;

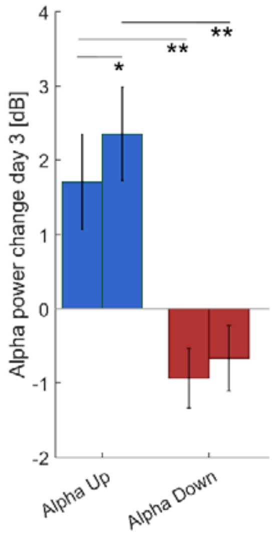

Group		Alpha power day 3		*Mdiff*(*SEM*)	*t*(*df*)	*p*	95% Confidence interval	
							Upper	Lower
Alpha Up		NF block 1	NF block 2	-.64(.21)	-3.06(16)	.008*	-1.091	-.197
Alpha Down		NF block 1	NF block 2	-.27(.32)	-.84(14)	.415	-.954	.417
Alpha Up	Alpha Down	NF block 1	NF block 1	2.65(.78)	3.39(30)	.002**	1.051	4.238
Alpha Up	Alpha Down	NF block 2	NF block 2	3.02(.79)	3.83(30)	.001**	1.411	4.628

*Note. *The level of significance (*p < .05; *** p *< .01) was obtained after Bonferroni adjustment (.05/4 =.013; .001/4 = .003); Data are presented as mean ± SEM.*

Supplementary Table 4 | Post hoc tests – two-way mixed *ANOVA* 2 x 4 (time x condition) comparing tactile acuity pre and post measures of all groups;

Tactile acuity day 3	Group		*Mdiff*(*SEM*)	*t*(*df*)	*p*	95% Confidence interval	
						Upper	Lower
Pre	Alpha Up	Alpha Down	.13(.08)	1.73(30)	.094	-.023	.285
		Control	.11(.07)	1.47(35)	.152	-.041	.257
		NF-PR	.16(.08)	1.93(31)	.063	-.009	.322
	Alpha Down	Control	-.02(.07)	-.33(33)	.743	-.166	.119
		NF-PR	.03(.08)	.33(29)	.743	-.133	.184
	Control	NF-PR	.05(.08)	.65(34)	.520	-.104	.202
Post	Alpha Up	Alpha Down	-.17(.08)	-2.21(30)	.035	-.331	-.013
		Control	-.01(.08)	-.07(35)	.946	-.174	.163
		NF-PR	-.02(.07)	-.27(31)	.789	-.172	.132
	Alpha Down	Control	.17(.09)	1.93(33)	.061	-.008	.342
		NF-PR	.15(.08)	2.01(29)	.054	-.003	3.07
	Control	NF-PR	-.01(.08)	-.18(34)	.862	-.182	.153
Tactile acuity day 3		Group	*Mdiff*(*SEM*)	*t*(*df*)	*p*	95% Confidence interval	
						Upper	Lower
Pre	Post	Alpha Up	.25(.02)	11.07(16)	.000***	.295	.303
		Alpha Down	-.05(.03)	-1,62(14)	.129	-.115	.016
		Control	.14(.04)	3.75(19)	.001*	.062	.219
		NF-PR	.08(.04)	2.10(15)	.053	-.001	.156

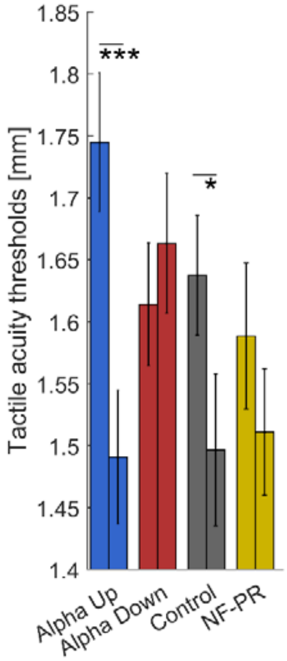

*Note. *The level of significance (*p < .05; ** p < .01; *** p <* .001*) was obtained after Bonferroni adjustment (.05/16 =.003; .01/16 = .0006; .001/9 = .00006); Data are presented as mean ± SEM.*

Supplementary Table 5 | LSD Fisher post hoc test for comparisons of tactile acuity gains between all conditions

Group		*Mdiff*(*SEM*)	*t*(*df*)	*p*	95% Confidence interval	
					Upper	Lower
Alpha Up	Alpha Down	17.84(3.02)	1.73(30)	.000***	11.80	.285
	Control	5.92(2.82)	1.47(35)	.039*	0.30	.257
	NF-PR	10.42(2.97)	1.93(31)	.001***	4.48	.322
Alpha Down	Control	-11.92(2.92)	-.33(33)	.000***	-17.74	-6.09
	NF-PR	-7.41(3.07)	.33(29)	.019*	-13.54	-1.29
Control	NF-PR	4.50(2.86)	.65(34)	.121	-1.22	10.22

Note. * *p* < .05; ** *p* < .01; *** *p* < .001; Data are presented as mean ± SEM.

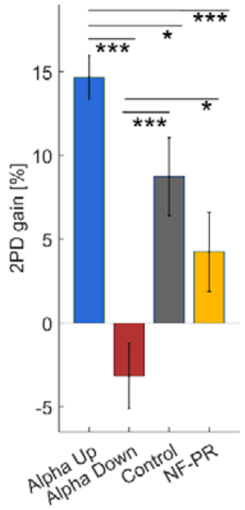

Supplementary Table 6 | Post hoc tests – Two-way mixed *ANOVA* 4 x 2 (time x condition) with alpha power during inter-train intervals of repetitive sensory stimulation, normalized on baseline day 1;

Group		Alpha power RSS	*Mdiff*(*SEM*)	*t*(*df*)	*p*	95% Confidence interval	
						Upper	Lower
Alpha Up	Alpha Down	0 - 5 min	2.51(.89)	2.82(22)	.010*	.667	4.364
		5 - 10 min	3.31(.93)	3.55(22)	.002**	1.376	5.247
		10 - 15 min	3.10(.88)	3.51(22)	.002**	1.270	4.931
		15 - 20 min	2.15(.84)	2.57(22)	.017	0.415	3.878

Note. *The level of significance (**p* < .05; ** *p* < .01) was obtained after Bonferroni adjustment (.05/4 =.013; .01/16 = .003); Data are presented as mean ± SEM.

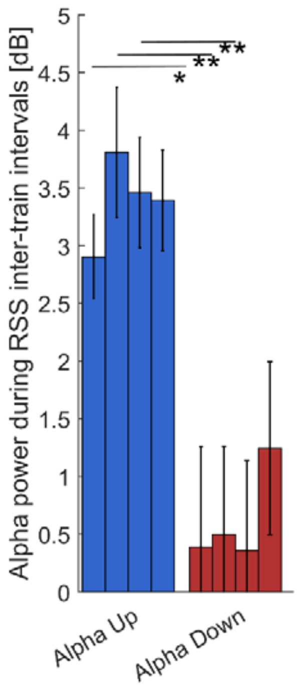

Supplement: Supplementary file 1 — Supplementary Tables [file 41467_2018_8012_MOESM1_ESM.pdf]
